# EpCAM Intracellular Domain Promotes Porcine Cell Reprogramming by Upregulation of Pluripotent Gene Expression via Beta-catenin Signaling

**DOI:** 10.1038/srep46315

**Published:** 2017-04-10

**Authors:** Tong Yu, Yangyang Ma, Huayan Wang

**Affiliations:** 1 Department of Animal Biotechnology, College of Veterinary Medicine, Northwest A&F University, Yangling, Shaanxi 712100, China

## Abstract

Previous study showed that expression of epithelial cell adhesion molecule (EpCAM) was significantly upregulated in porcine induced pluripotent stem cells (piPSCs). However, the regulatory mechanism and the downstream target genes of EpCAM were not well investigated. In this study, we found that *EpCAM* was undetectable in fibroblasts, but highly expressed in piPSCs. Promoter of *EpCAM* was upregulated by zygotic activated factors LIN28, and ESRRB, but repressed by maternal factors OCT4 and SOX2. Knocking down *EpCAM* by shRNA significantly reduced the pluripotent gene expression. Conversely, overexpression of EpCAM significantly increased the number of alkaline phosphatase positive colonies and elevated the expression of endogenous pluripotent genes. As a key surface-to-nucleus factor, EpCAM releases its intercellular domain (EpICD) by a two-step proteolytic processing sequentially. Blocking the proteolytic processing by inhibitors TAPI-1 and DAPT could reduce the intracellular level of EpICD and lower expressions of OCT4, SOX2, LIN28, and ESRRB. We noticed that increasing intracellular EpICD only was unable to improve activity of EpCAM targeted genes, but by blocking GSK-3 signaling and stabilizing beta-catenin signaling, EpICD could then significantly stimulate the promoter activity. These results showed that EpCAM intracellular domain required beta-catenin signaling to enhance porcine cell reprogramming.

The generation of porcine pluripotent stem cells may not only prove the concept of pluripotency in domestic animals, but also retain the enormous potential for animal reproduction and translational medicine. In last several years, porcine induced pluripotent stem cells (piPSCs) were generated in many research groups including our laboratory^1^,^2^,^3^,^4^,^5^,^6^,^7^,^8^. Because *bona fide* pig embryonic stem cells were not available yet, most of manipulation conditions for maintenance of piPSCs were consulted with the conditions for mouse iPS^9^ and human iPS cells^10^. Therefore, the reported piPSCs showed the divers morphology and biological features. Some piPSC lines were bFGF-dependent and showed mouse epiblast-derived stem cell like morphology^2^,^11^; other lines were LIF-dependence and showed mouse ESC-like morphology^3^. Thus, the optimal culture condition and regulatory circuitry for generation and maintenance of piPSCs are not standardized, and the generation and maintenance of naïve state piPSCs is still an important issue that has to be addressed. Previous reports were sure that signaling pathways used for maintaining human and mouse iPSCs did not sustain the self-renewal and pluripotency of porcine iPSCs^12^,^13^. The species-related regulatory signaling pathway as reported in mouse and human pluripotent stem cells (PSCs)^14^ is likely to be applied in pig and other animals, in which PI3K/AKT signaling and TGF-beta signaling pathways, instead of LIF and bFGF signaling pathways, may play key roles to maintain porcine stem cell pluripotency^15^.

The epithelial cell adhesion molecule (EpCAM) is a transmembrane glycoprotein encoded by the *TACSTD1* gene, and is highly expressed in epithelia and epithelial-derived neoplasms^16^. In human and mouse iPSCs, EpCAM was also highly expressed and play a critical role in cell reprogramming^17^,^18^,^19^,^20^. Consistently, our previous study showed that *EpCAM* is highly expressed in porcine iPSCs^13^. Therefore, as a cell-to-cell adhesion molecule, EpCAM is involved in cell signaling, migration, proliferation, and differentiation^19^,^20^,^21^. Recent studies showed that EpCAM was a key surface receptor that was able to translocate to the nucleus and to regulate downstream target gene expression^22^. Through two-step proteolytic processing, EpCAM is sequentially cleaved by tumor necrosis factor-alpha converting enzyme (TACE/ADAM17) and presenilin 2 (PS-2), a protease component of gamma-secretase complex, and releases an N-terminal extracellular domain (EpEX) and a 5 kDa C-terminal intracellular domain (EpICD). The EpICD fragment, which is unstable in the cytoplasm, is able to translocate into nucleus and comes along with co-transcriptional activators to stimulate gene expression and cell proliferation^23^. The study showed that EpICD with FHL2, beta-catenin, and Lef-1 formed a nuclear complex, which contacted DNA at Lef-1 consensus sites, and stimulated *c-Myc* expression^24^. Consequently, the role of EpCAM in porcine cell proliferation and its association with reprogramming is worth to be investigated.

Studies have shown the fundamental function of EpCAM in regulation of human and mouse pluripotent stem cells^17^,^18^. In order to gain insight into the epigenetic regulation of porcine pluripotency, we comprehensively analyzed porcine EpCAM gene and investigated the regulation function of EpCAM for porcine cell reprogramming and maintenance of pluripotency. Our discoveries would be conducive to establish naïve state of porcine pluripotent stem cells.

## Results

### EpCAM Is Highly Expressed in Porcine Pluripotent Stem Cells

**Figure 1:**
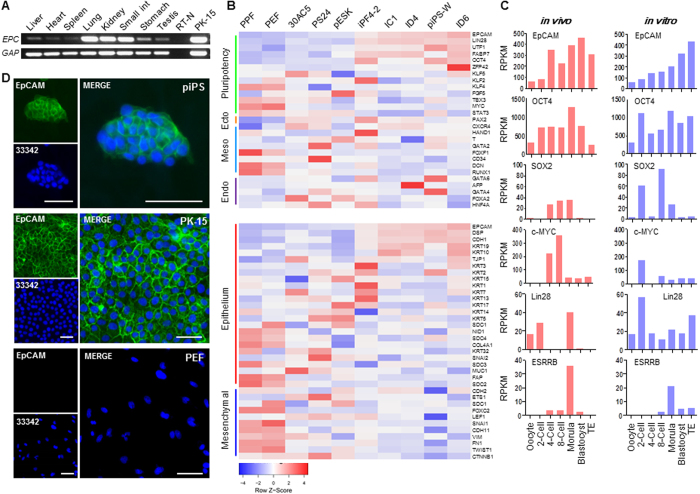
EpCAM expression in porcine tissues and iPSCs. (**A**) *EpCAM (EPC*) expression in porcine tissues and epithelial cell PK-15. *GAPDH (GAP*) was used as internal control. (**B**) Expression profile of vital genes for the pluripotency and three germ layers (upper), and MET/EMT markers (lower) in porcine somatic and pluripotent cells. (**C**) Expression profile of *EpCAM* and pluripotent genes in porcine oocytes and early stage embryos maturated *in vivo* and *in vitro*. (**D**) Immunofluorescences of EpCAM (green) in piPS, PK-15, and PEF cells. Nuclei were stained with Hoechst 33342 (blue). Scale bar, 50 μm.

The expression profile of *EpCAM* in porcine tissues from newborn piglet was conducted by RT-PCR analysis. As described previously^25^,^26^, EpCAM is highly expressed in epithelial cells. In our study, *EpCAM* message was detectable in all tested samples, which may be due to the widespread epithelial cells in most of organs. In those epithelia enriched organs, for instance lung, kidney, and small intestine, EpCAM was relatively abundant than in other tissues (Fig. 1A). The heatmap of microarray data (note: *NANOG* and *SOX2* genes were not included in the Affymetrix Pig GeneChipe^13^) of eight piPSC lines and two primary porcine skin fibroblasts showed that *EpCAM* and core pluripotent genes, such as *OCT4, LIN28, UTF1*, and *ZFP42*, were highly expressed, and the expression of lineage-specific genes were downregulated (Fig. 1B), suggesting that *EpCAM* might play an important role during porcine cell reprogramming. Additionally, the expression level of *EpCAM* and many epithelial genes were clearly higher in piPSCs than in fibroblasts, and the expression level of mesenchymal markers was clearly downregulated in piPSCs (Fig. 1B). These results indicated that a mesenchymal to epithelial transition was occurred during the porcine cell reprogramming, which was an important event during cell reprogramming as reported in mouse and human iPSCs^27^,^28^.

Since the inner cell mass (ICM) derived embryo stem cells (ESCs) are the most extensively studied pluripotent stem cells^29^, transcriptome data of porcine oocyte and early state embryos maturated *in vivo* and *in vitro* were analyzed. The level of *EpCAM* expression was low in oocyte and 2-cell stages, but significantly increased in 4/8-cell stage till blastocyst stage (Fig. 1C), suggesting that *EpCAM* is a zygotic activated gene. We noticed that the high level expression of zygotic activated pluripotent genes *LIN28*^30^ and *ESRRB*^31^ was also presented at the morula stage in *in vivo* embryos though the expression profile of pluripotent genes displayed the slightly different expression patterns in *in vitro* embryos. Being consistent with the transcriptomic and RT-PCR analyses, immunofluorescence staining confirmed that EpCAM protein was expressed and translocated to the cellular membrane in piPSCs, but was undetectable in non-reprogrammed PEF cells (Fig. 1D). These results revealed that EpCAM could be a marker to monitor porcine somatic cell reprogramming.

### EpCAM Is Activated by Zygotic Activated Pluripotent Factors

**Figure 2:**
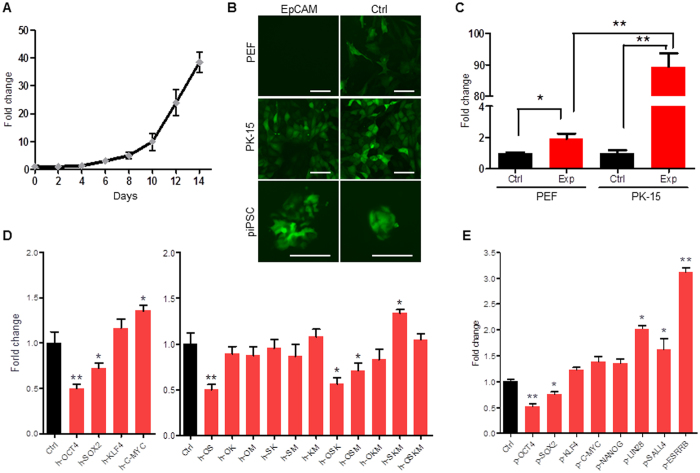
EpCAM promoter activation in reprogrammed cells . (**A**) The qRT-PCR analysis of *EpCAM* expression during porcine fibroblast reprogramming by hOSKM for 14 days. (**B**) Transduction of pTRIP-EpCAM-EGFP and pTRIP-CAGG-EGFP (Ctrl) into PEF, PK-15, and piPS cells, respectively, to determine activation of the EpCAM promoter. Scale bar, 100 μm. (**C**) Luciferase assay. Reporter pGL3-EpCAM-Pro (Exp) and pGL3-Basic (Ctrl) were transfected into PEF and PK-15 cells, respectively, for 36 h. (**D**) Luciferase assay of EpCAM promoter activation regulated by human pluripotent factor alone (left), or combination (right) in 293 T cells. Ctrl, cells were transfected with pGL3-EpCAM-Pro and pMXs-EGFP. (**E**) Luciferase assay of EpCAM promoter activation that was regulated by porcine pluripotent factors. Data are presented as mean ± S.D., *P < 0.05, **P < 0.01, n = 3.

To further investigate the regulation of EpCAM in piPSCs, we detected the expression of *EpCAM* during porcine cell reprogramming. In the first four days post transfection of human *OCT4, SOX2, KLF4*, and *c-MYC* (hOSKM), the endogenous *EpCAM* was at very low level. After six days post transfection, *EpCAM* expression was significantly increased (Fig. 2A). To explore how *EpCAM* was activated, a 3.8 kb DNA fragment (GenBank accession number KY218795) that contains promoter region, 5′ UTR sequence, and ATG codon of *EpCAM* was cloned, confirmed by DNA sequencing, and subcloned into reporter vectors pTRIP-EGFP and pGL3-basic to form pTRIP-EpCAM-EGFP and pGL3-EpCAM-Pro constructs (Table S2). Results showed that a strong EGFP fluorescence was observed in PK-15 and piPSC cells, but the fluorescent signal was undetectable in control PEF cells (Fig. 2B). Luciferase assay showed that *EpCAM* promoter was significantly activated in epithelia PK-15 versus fibroblasts PEF (Fig. 2C), indicating the cell-specificity of cloned *EpCAM* promoter.

To investigate whether *EpCAM* was directly regulated by pluripotent factors, the reporter pGL3-EpCAM-Pro with hOSKM vectors were co-transfected into 293 T cells, respectively. We noticed that *EpCAM* promoter activity was significantly repressed by OCT4 and SOX2 (Fig. 2D). Since OCT4 and SOX2 can form a complex, bind to downstream target genes, and maintain the pluripotency^32^, the multiple transduction of OSKM factors were applied. Results showed that OCT4-SOX2 complex as well as OSK and OSM combinants could significantly reduce *EpCAM* promoter activity, but combinations of OCT4-KLF4, OCT4-c-MYC, SOX2-KLF4, SOX2-c-MYC, and KLF4-c-MYC did not influence *EpCAM* activity. However, c-MYC and SKM combinant were able to enhance *EpCAM* promoter activity (Fig. 2D). We then detected whether *EpCAM* could be regulated by pig-oriented pluripotent factors. Similar to human factors, porcine OCT4 and SOX2 repressed *EpCAM* promoter activity (Fig. 2E). Additionally, zygotic activated pluripotent factors LIN28 and ESRRB could significantly upregulate *EpCAM* activity, suggesting that *EpCAM* is upregulated by zygotic activated pluripotent factors.

### Knockdown EpCAM Reduces the Reprogramming Efficiency

**Figure 3:**
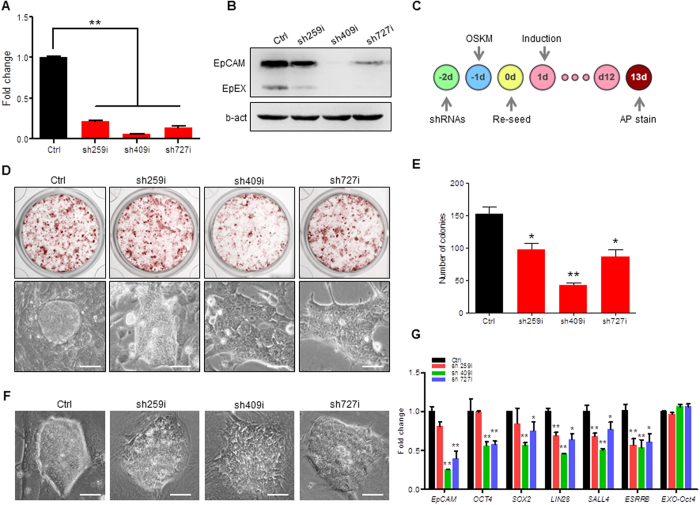
Knockdown EpCAM expression in piPSCs. (**A,B**) Quantitative RT-PCR (**A**) and western blotting (**B**) analyses of EpCAM expression knockdown by shRNAs in PK-15 cells for 60 h. b-act, beta-actin was used as internal control. (**C**) Schematic diagram of cell reprogramming by hOSKM and shRNA treatment. (**D**) Alkaline phosphatase staining of cells that were reprogrammed with shRNAs for 13 days (upper) and the morphology of reprogrammed cell colony (lower). (**E**) The number of AP positive colonies after shRNA treatment for 13 days. (**F**) Morphology of piPSCs that were transfected with shRNA. (**G**) qRT-PCR analysis of pluripotent gene expressions. DOX-iPSCs were transfected with shRNAs for 60 h. Ctrl, cells were treated without shRNA. Scale bar, 50 μm. Data are presented as mean ± S.D., *P < 0.05, **P < 0.01, n = 3.

To knock down EpCAM expression, we designed three shRNAs that targeted to EpCAM extracellular domain sequence, and constructed three lentiviral shRNA vectors (Fig. S1). When PK-15 cells were transfected by lentiviral shRNA, the expression of endogenous EpCAM was down 70–90 percent compared to the control in both RNA level (Fig. 3A) and protein level (Fig. 3B). A diagram of cell reprogramming by four factors hOSKM and shRNA treatments was illustrated in Fig. 3C. PEF cells that were transfected with lentiviral shRNAs were reprogrammed by hOSKM (Fig. 3C). For two weeks induction and shRNA treatment, the reprogrammed cells showed the alkaline phosphatase (AP) staining positive colonies (Fig. 3D, upper), and the morphology of EpCAM knockdown cells showed much incompact (Fig. 3D lower). The quantification of AP-positive colonies showed that the reprogramming efficiency was significantly reduced when *EpCAM* expression was knocked down by shRNA (Fig. 3E). We then detected the effect of EpCAM in piPSCs. The shRNA constructs were transfected into DOX-iPSCs, a DOX induced piPSC line described previously^11^, for 60 h, respectively. The morphology of shRNA treated iPSC clones presented much looser and flat comparing to control clones (Fig. 3F). Quantitative RT-PCR analysis showed that in EpCAM-knockdown cells, the expression level of pluripotent genes including maternal factors *OCT4, Sox2*, and *SALL4* and zygote activated factors *LIN28* and *ESRRB* were significantly decreased (Fig. 3G), suggesting that EpCAM as an key surface-to-nucleus factor plays an important role to regulate porcine pluripotent gene expression.

### Overexpression of EpCAM Enhances Reprogramming Efficiency

**Figure 4:**
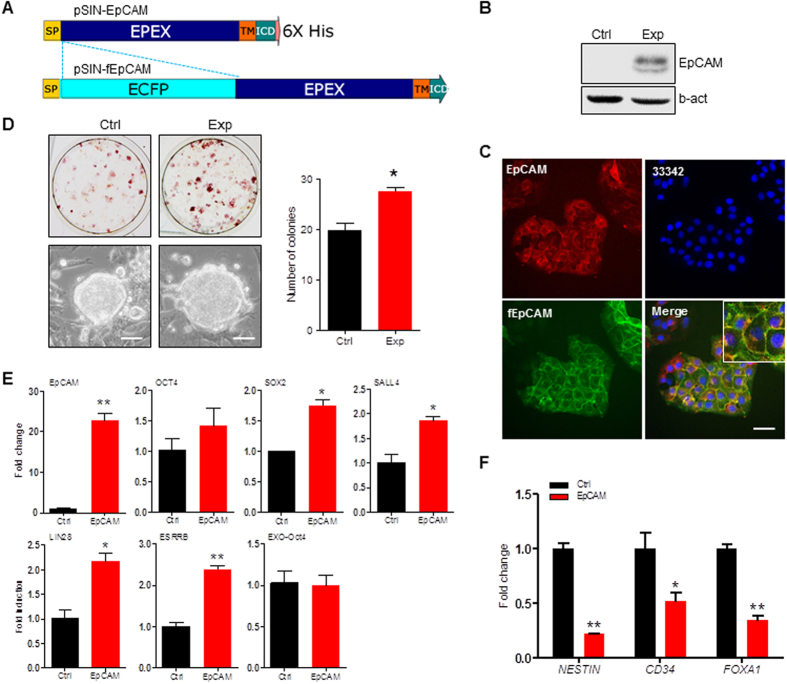
Overexpression of EpCAM improves PEF cell reprogramming. (**A**) Schematic diagram of lentiviral vectors for expressing EpCAM and ECFP-EpCAM fusion protein (fEpCAM). SP, signal peptide. TM, transmembrane domain. EpEX, extracellular domain. ICD, intracellular domain. (**B**) Western blotting analysis. Proteins were prepared from PEF (Ctrl) and PEF + EpCAM (Exp) cells that were transfected by pSIN-EpCAM lentivirus for 60 h. Anti-EpCAM antibody and anti-b-actin antibody were applied for immunoblotting. (**C**) Immunostaining of EpCAM by anti-EpCAM antibody (red) in PK-15 cells that were transfected with pSIN-fEpCAM (green). Nuclei were stained with Hoechst 33342 (blue). Scale bar, 25 μm. (**D**) AP staining of cells reprogrammed by OSKM for 13 days (upper) and the morphology of derived colonies (lower). AP positive colonies were counted (right panel). Scale bar, 50 μm. (**E**) qRT-PCR analysis of pluripotent gene expressions in DOX-iPSCs. Ctrl, cells were transfected with pSIN-EGFP. Exp, cells were transfected with pSIN-EpCAM. (**F**) qRT-PCR analysis of gene markers in piPS cells that were spontaneously differentiated into ectoderm (*NESTIN*), mesoderm (*CD34*), and endoderm (*FOXA1*). Data are presented as mean ± S.D., *P < 0.05, **P < 0.01, n = 3.

To further investigate EpCAM regulation function, we cloned porcine EpCAM coding DNA sequence (GenBank Accession No. KX904866), and constructed two overexpression vectors. The construct pSIN-EpCAM was derived from pSIN lentiviral plasmid with puromycin resistance, and the construct pSIN-fEpCAM was derived from pSIN-EpCAM with ECFP (enhanced cyan fluorescent protein) insertion in N-terminal of EpCAM protein (Fig. 4A). Western blotting analysis of PEF cells that were stably transfected by pSIN-EpCAM and selected by puromycin showed that cloned gene encoded EpCAM protein and overexpressed in transfected PEF cells (Fig. 4B). We then transfected pSIN-fEpCAM into PK-15 cells, and did immunofluorescence staining, which showed that fEpCAM fusion protein and endogenous EpCAM were co-localized in cell plasma membrane (Fig. 4C), indicating that the cloned EpCAM retained the biological function, and the constructs were able to be used for the following experiments.

To determine EpCAM regulation during cell reprogramming, the pSIN-EpCAM was transfected into PEF cells, which were then induced by retroviral vectors carrying with hOSKM. For 13 days induction, the compact colonies were formed (Fig. 4D, lower) that showed AP-positive staining (Fig. 4D, upper). A quantification result showed that overexpression of EpCAM significantly increased the number of AP-positive colonies compared to the control group (Fig. 4D). Overexpression of EpCAM in DOX-iPSCs, which were maintained by addition of doxycycline, could also significantly elevate the expression of endogenous pluripotent genes, including *SOX2, SALL4, LIN28*, and *ESRRB* (Fig. 4E). We noticed that *OCT4* expression was increased slightly, but not statistically significant, which may be due to the existence of high level DOX-induced OCT4 in DOX-iPSCs. We also detected the differentiation potential of piPS cells that were over expressed EpCAM. Results showed that overexpression of EpCAM maintained the self-renewal of piPS cells and impede cells to differentiate into the three germ layers (Fig. 4F).

### EpCAM Cleavage and EpICD Generation

**Figure 5:**
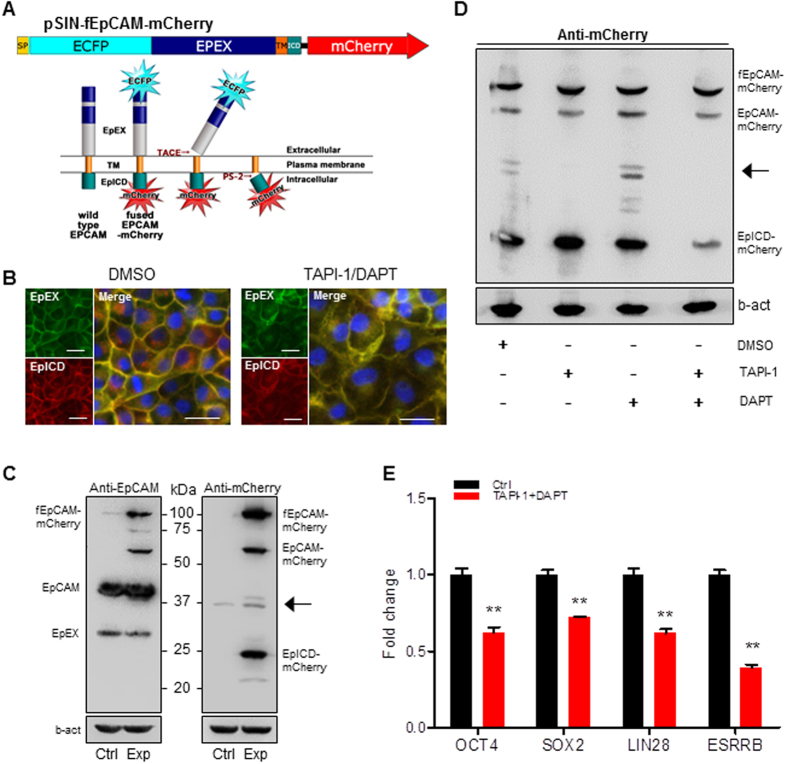
EpCAM cleavage and EpICD generation. (**A**) Schematic diagram of the lentiviral vector pSIN-fEPCAM-mCherry carrying with ECFP/mCherry double labeled EpCAM. The functional domains EpEX and EpICD can be released sequentially by TACE and PS-2. (**B**) Florescent images of EpEX (green) and EpICD (red) in PK-15 cells, which were transfected by pSIN-fEpCAM-mCherry and treated with or without TAPI-1/DAPT. Nuclei were stained by Hoechst 33342 (blue). Scale bar, 25 μm. (**C**) Western blotting was performed by anti-EpCAM and anti-mCherry antibodies, respectively. PK-15 cells were transfected by pSIN-EGFP (Ctrl) and pSIN-fEpCAM-mCherry (Exp), respectively. (**D**) Western blotting was performed by anti-mCherry antibody with the treatment of TAPI-1, DAPT, and DMSO in PK-15 cells. b-actin (b-act) was as internal control. The arrow indicates the partial degradation of extracellular domain fraction. (**E**) qRT-PCR analysis of pluripotent gene expression in piPSCs with or without addition of TAPI-1 and DAPT. Ctrl, cells were treated with DMSO. Data are presented as mean ± S.D., *p < 0.05, **p < 0.01, n = 3.

To monitor EpCAM protein processing, the protein was double labeled with ECFP in N-terminal and mCherry in C-terminal. The N-terminal domain EpEX (green) and C-terminal domain EpICD (red) can be cleavage sequentially by TACE/ADAM17 and PS-2 (Fig. 5A). The pSIN-fEpCAM-mCherry vector was transfected into pig (PEF and PK15), mouse (NIH3T3 and P19), and human (293 T and Hela) cells, respectively. The fEpCAM-mCherry fusion protein was highly expressed in epithelial and pluripotent cells, but low expression in fibroblasts (Fig. S2). Results of transfection of pSIN-fEpCAM-mCherry into PK-15 cells showed that EpICD (red) was released from cytoplasm membrane and translocated to nuclei in control (DMSO) cells. However, the cleavage of EpICD was blocked by addition of protease inhibitors TAPI-1 and DAPT since the fEpCAM-mCherry fusion protein was remained in cytoplasm membrane (yellow) (Fig. 5B). Western blotting assay showed that endogenous EpCAM and ectopically expressed fEpCAM-mCherry were detected by anti-EpCAM antibody presenting an approximate 39 kDa protein band of matured EpCAM and EpEX, and the ectopically expressed EpICD-mCherry was detected by anti-mCherry antibody (Fig. 5C). When fEpCAM-mCherry expressed PK-15 cells were treated with gamma-secretase complex inhibitor DAPT, the EpICD fraction was decreased and the partially degraded extracellular domain fraction was accumulated, which was undetectable in the treatment of TAPI-1, a TACE/ADAM17 inhibitor that prevents proteolytic cleavage of the EpCAM extracellular domain. However, when application of both TAPI-1 and DAPT, the level of EpICD in nuclear extract was significantly reduced, indicating that both factors block the cleavage of EpCAM (Fig. 5D). We then investigated the cleavage and function of EpICD in porcine iPS cells. Results of addition of TAPI-1 and DAPT in DOX-iPSCs cells showed that expression of pluripotent factors, including *OCT4, SOX2, LIN28*, and *ESRRB*, was significantly reduced (Fig. 5E), showing the similar result that EpCAM expression was knocked down by shRNAs (Fig. 3G). This observation indicates that EpICD is a core transcription activator to upregulate expression of porcine pluripotent factors.

### EpICD Regulates Pluripotent Gene Expression Requiring beta-catenin Signaling

**Figure 6:**
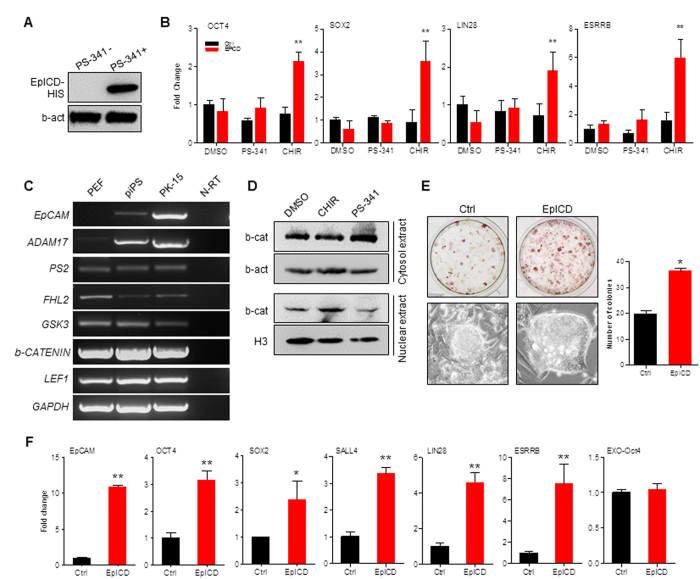
EpICD regulates cell reprogramming through b-catenin signaling. (**A**) Western blotting of EpICD expression in PEFs with or without proteasome inhibitor PS-341. (**B**) Luciferase assay. The pSIN-EpICD with pGL3-OCT4-Pro, pGL3-SOX2-Pro, pGL3-LIN28-Pro, and pGL3-ESRRB-Pro were co-transfected into PEFs for 36 h in medium with PS-341 and CHRI99021 (CHIR), respectively. DMSO was used as control. (**C**) RT-PCR analysis of genes associated with EpCAM signaling. *GAPDH* was used as control. (**D**) Western blotting of b-catenin (b-cat) protein in cytoplasm and nucleus. PEF cells were treated with DMSO, CHRI99021 (CHIR) and PS-341, respectively. Beta-actin (b-act) and histone H3 (H3) were determined as internal controls. (**E**) PEF cells were reprogrammed by hOSKM for 13 days with (Ctrl) or without (EpICD) co-transfection of pSIN-EpICD. AP staining (upper), the morphology of derived colonies (lower), and counts of AP positive colonies (right panel). (**F**) qRT-PCR analysis of pluripotent gene expressions in piPSCs, which were transfected by pSIN-EpICD. Ctrl, cells were transfected by pSIN-EGFP. Data are presented as mean ± S.D., *p < 0.05, n = 3, **p < 0.01, n = 3.

Because the size of EpICD-mCherry fusion protein is extremely larger than EpICD and causes the difficulty to translocation into nuclei, as seen in Fig. S2 that EpICD-mCherry was accumulated around nucleus (Figs S2, 5B), we then constructed pSIN-EpICD vector that contained an EpICD peptide fused with His tag. As previously reported that EpICD could be degraded by proteasome^33^, the proteasome inhibitor PS-341 was used when overexpression of EpICD in PEF, in which most of ectopically expressed EpICD was degraded if without addition of PS-341 (Fig. 6A), indicating that EpICD was unstable in PEF cells. To determine EpICD regulation function to enhance the transcriptional activation, promoter sequences of *OCT4* (4.2 kb, KY218798), *SOX2* (3.0 kb, KY218799), *LIN28* (3.3 kb, KY218797), and *ESRRB* (3.3 kb, KY218796) were subcloned into pGL3-Basic reporter vector. When pSIN-EpICD was co-transfected with reporter vectors into PEFs, the promoter activity was not activated. To avoid EpICD degradation by proteasome, the inhibitor PS-341 was added in culture medium to elevate EpICD peptide level, but the promoter activity was still not activated (Fig. 6B), suggesting that quantity of EpICD was not essential for the activation of pluripotent genes. We then added inhibitor CHIR99021 into medium, which inhibits GSK-3 activity and therefore stabilize intracellular beta-catenin^34^. Results of promoter activity showed that expressions of *OCT4* (2.8 fold), *SOX2* (4.0 fold), *LIN28* (2.6 fold), and *ESRRB* (3.4 fold) were significantly increased (Fig. 6B), indicating that EpICD required beta-catenin signaling to stimulate pluripotent gene expression. We then investigated EpCAM and beta-catenin signaling associated gene expressions in fibroblast, epithelia, and piPS cells. Except for *EpCAM* and a regulated intramembrane proteolysis initiator gene *ADAM17*, which were very lowly expressed in PEFs, the other regulated intramembrane proteolysis initiator gene *PS2*, the ICD binding partner gene *FHL2, GSK3, beta-Catenin*, and *LEF1*, an enhancer-binding factor formed a complex with beta-Catenin, were stably expressed in all three cell lines (Fig. 6C). This observation suggested that different cleavage patterns and signaling pathways might exist among cell types. The western blotting assay confirmed that there was more active beta-catenin in nuclear fraction when GSK3 inhibitor CHIR99021 was applied in PEFs, while the protein level of inactive beta-catenin in cytosol was basically consistent among three treatments (Fig. 6D). These results pointed out that GSK3 inhibitor CHIR99021 might be required as an essential ingredient in medium to maintain porcine iPSC self-renewal.

To confirm whether EpICD and CHIR99021 could enhance cell reprogramming, PEFs were induced by retroviral vectors carrying with hOSKM and EpICD for 13 days in the medium supplemented with CHIR99021. The AP staining and the number of AP-positive colonies were significantly increased in EpICD-expressed group than in control group (Fig. 6E). Quantitative RT-PCR analysis showed that EpICD significantly increased endogenous pluripotent gene expression including *OCT4* (3.2 fold), *SOX2* (2.4 fold), *SALL4* (3.3 fold), *LIN28* (4.6 fold), and *ESRRB* (7.5 fold) (Fig. 6F).

**Figure 7:**
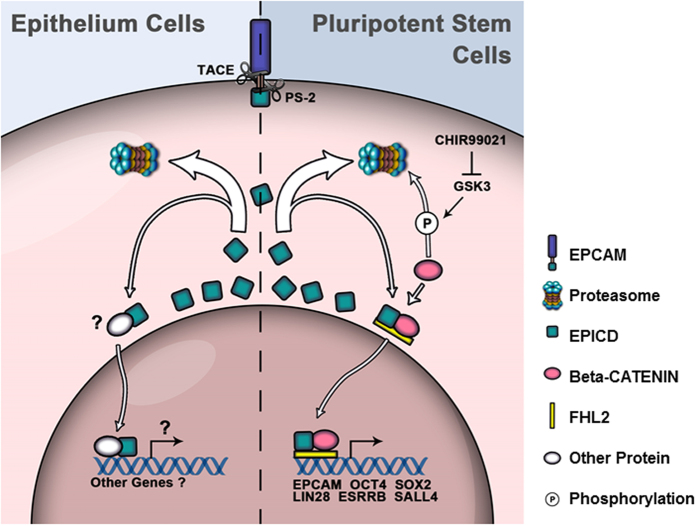
Diagram of EpCAM signaling in epithelial cells and pluripotent stem cells.

Based on this study and other laboratory’s reports^24^,^35^, we indicate that in epithelial cells EpICD molecule released from EpCAM is immediately removed by proteasome degradation, while in reprogrammed cells EpICD with beta-catenin and FHL2 forms a complex, translocates into nucleus, and subsequently promotes downstream pluripotent gene expression (Fig. 7).

## Discussion

As a single-transmembrane glycoprotein, EpCAM is one core tumor-associated antigens^36^ and is the diagnosis and therapeutic target for various cancers^37^. However, recent study showed that high-level expression of EpCAM was required for the maintenance of self-renewal in mouse ES cells, especially under the differentiation condition in the absence of LIF, ectopic expression of EpCAM could partially compensate and retain ES phenotype^33^. Further study showed that in partially reprogrammed stem cells EpCAM did not activated, but in fully reprogrammed mouse iPSC cells EpCAM was highly expressed, indicating that EpCAM could be identified as a new surface marker for efficiently identifying fully reprogrammed iPSCs^20^. High-level and selective expression of EpCAM was also reported in undifferentiated, rather than differentiated, hES cells^18^. When knocked down endogenous EpCAM, hESC decreased cell growth and increased gene expression in the endoderm and mesoderm lineages^19^. In our previously study, we found that no matter what induction conditions were used, *EpCAM* was upregulated and highly expressed in porcine iPSCs^13^. Further analysis of transcriptome data from early state embryos maturated *in vivo* and *in vitro* showed that *EpCAM* expression was low in oocyte and 2-cell stages, but significantly increased in 4/8-cell stage (Fig. 1), which is the time of the maternal to zygotic transition in porcine embryogenesis^38^,^39^. We found in this study that the expression level of *EpCAM* was correlated with the expression of pluripotent genes. Down regulation of *EpCAM* expression by shRNA interference or ectopic expression EpCAM could significantly influence endogenous pluripotent gene expression and the formation of AP-positive colonies (Figs 3 and 4). In accord with the findings in mouse and human^18^,^20^, we confirmed that in OSKM mediated induced pluripotent stem cells EpCAM could be a critical marker that monitors pluripotent state of reprogrammed cells.

Studies in mouse and human pluripotent stem cells showed that EpCAM and its intercellular domain could directly regulate the promoter activation of pluripotent genes including Oct4, Sox2, and Nanog etc.^17^,^18^. In porcine iPS cells, overexpression of EpCAM significantly enhanced the promoter activation of *OCT4* and *SOX2*. Furthermore, the promoter activity of *LIN28*, which is used as one of the reprogramming factors in hiPSCs^10^ and is an essential factor of nucleologenesis during early embryonic development^30^, and ESRRB, which plays the key role in regulating the naïve pluripotent state^40^, were also significantly stimulated by EpCAM. On the other hand, the activation of EpCAM promoter was also regulated by pluripotent factors. We noticed that zygotic activated pluripotent factors LIN28^30^ and ESRRB^40^ significantly stimulated *EpCAM* promoter activation. Unexpectedly, the maternal pluripotent factors OCT4 and SOX2, as well as OS, OSK, and OSM combinants, significantly suppressed *EpCAM* promoter activation. This founding indicates that as a zygotic activated gene EpCAM with other zygotic activated pluripotent factors forms a positive feedback regulatory circuitry to activate pluripotent gene expression and to maintain pluripotency of iPS cells. In hOSKM mediated PEF reprogramming, EpCAM expression did not immediately upregulated, but was delayed till 6 days post induction. This observation indicated that EpCAM could be used as a reprogramming selection marker to monitor porcine cell reprogramming.

By two-step proteolytic processing through adequate cell-to-cell contact EpCAM is sequentially cleaved and plays the function of surface-to-nucleus signaling^22^. Upon TACE/ADAM17 cleavage, the extracellular domain EpEX is released as a soluble ligand, and gamma-secretase complex cleavage releases the C-terminal intracellular domain EpICD, which is in combination with four-and-a-half LIM domains protein 2 and beta-catenin and drives cell proliferation^24^. We found that EpICD fragment was unstable in the cytoplasm due to the proteasome degradation; however, EpICD degradation could be diminished by application of the proteasome inhibitor (Fig. 6). This observation might partially explain why the high level endogenous EpCAM found in epithelial cells did not activate pluripotent gene expression.

The signaling pathway of EpCAM includes members of EpEX, EpICD, E-cadherin, Cldn7, beta-catenin, presenilin-2 (PS2), and ADAM17^17^,^41^. We found that by just increasing the quantity of EpICD in cytoplasm, EpCAM signaling was not functional because the promoter of EpCAM downstream target genes were not activated (Fig. 6). However, when stabilizing the intracellular beta-catenin by blocking GSK-3 activity, EpCAM signaling could promote the activation of pluripotent genes *OCT4, SOX2, LIN28*, and *ESRRB* and enhance reprogramming efficiency. Thus, EpICD did require beta-catenin to play a role in the regulation of pluripotency of piPSCs, and preservation of Wnt/beta-catenin signaling by inhibition of GSK3 activity was necessitated with addition of CHIR99021 as an essential ingredient in culture medium. Furthermore, there were Tcf binding elements found in EpCAM promoter region. The Tcf/beta-catenin complex could bind to EpCAM promoter and regulate EpCAM gene expression, indicating that EpCAM is a Wnt/beta-catenin targeted gene^35^. Therefore, in addition to LIF and bFGF pathways, the Wnt/beta-catenin signaling pathway might be a key pluripotency-maintaining pathway for porcine pluripotent stem cells.

In summary, we confirmed that EpCAM was an important surface-to nucleus signaling molecule in piPSCs. Through releasing the intercellular domain, EpCAM complex proteins activated pluripotent gene expression and promoted cell reprogramming. This study is conducive to understand the regulatory mechanism of porcine cell reprogramming and to facilitate the generation of naïve porcine iPSCs.

## Methods

### Cell culture

Cell lines HEK-293, HEK-293T, Hela, PK-15 cells, and NIH3T3 cells were cultured in DMEM supplemented with 10% FBS, 1 mM L-glutamine (Gibco, USA), 0.1 mM nonessential amino acids (NEAA, Gibico, USA). Porcine embryonic fibroblast (PEF) and mouse embryonic fibroblast (MEF) cells were cultured in DMEM medium supplemented with 15% FBS. Mouse embryonic carcinoma cell P19 was cultured in α-MEM supplemented with 2.5% FBS. Cells were cultured at 37 °C with 5% CO_2_ in a humidified atmosphere. To maintain porcine DOX-iPSCs, a tetracycline operator (TetO)-inducible porcine induced pluripotent cell line reported in this laboratory previously^11^, cells were plated on MEF feeders, which were derived from C57 mice and mitotically inactivated, and cultured in an iPS medium consisted of DMEM, 15% FBS, 1mM L-glutamine, 0.1 mM NEAA, 0.1 mM β-mercaptoethanol, 10 ng/mL human LIF (LIF1050, Millipore), 10 ng/mL human basic FGF (100-18B, PeproTech), 3 μM CHIR99021 (S1263, Selleck Chemicals), 2 μM SB431542 (S1067, Selleck Chemicals), and 4 μg/mL doxycycline (D9891-25G-9, Sigma Aldrich) at 37 °C, 5% CO2. To inhibit protease activity, 50 μM TAPI-1 (an inhibitor of TACE/ADAM17, S7434, Selleck Chemicals), 50 μM DAPT (an inhibitor of the gamma-secretase complex, S2215, Selleck Chemicals), and 20 μM PS-341 (an inhibitor of proteasome, S1013, Selleck Chemicals) were applied in media where relevant experiment needs, respectively.

### RNA Extraction and RT-PCR

Total RNAs from porcine cells and tissues were extracted by Trizol Reagent (Invitrogen). RNAs were examined by measuring OD260/280 ratio, and samples with a ratio around 2.0 were used for reverse transcription. Two microgram total RNAs were reverse-transcribed with oligo-dT primer (Thermo Fisher) using RevertAid^TM^ reverse transcriptase (Thermo Fisher). RT-PCRs were performed using 2 × Es Taq MasterMix, 2 × Taq MasterMix (CW Biotech, China) for 32 cycles at 94 °C 30 s, 57 °C 30 s, and 72 °C 45 s. The negative control was done by directly performing PCR with total RNAs to check the contamination of genome DNA. Quantitative RT-PCRs (qRT-PCR) were performed using a 10-fold dilution of cDNA with SYBR Green PCR Master Mix (Takara Biotechnology, China), and detected with StepOnePlus Real-Time PCR System (Applied Biosystems). Reactions were performed for 40 cycles at 95 °C 15 s and 60 °C 60 s, and measurements were performed on three biological replicates and each reaction was performed in triplicate. The expression level of target gene was normalized to the expression level of *beta-actin*. Melting curve analysis was conducted to confirm the specificity. Primers used in this study are listed in Supplementary Table S1.

### Vector construction

To clone porcine EpCAM cDNA, total RNA was extracted from PK-15 cells. EpCAM coding DNA sequence (CDS) was amplified by RT-PCR using CloneAmp™ HiFi PCR Premix (#639298, Clontech). PCR fragment was cloned into pGEM-T Easy vector (A1360, Promega) and confirmed by DNA sequencing. Porcine EpCAM cDNA sequence was submitted to GenBank (Accession No. KX904866). To construct expression vector, EpCAM and EpICD fragments were subcloned into BamHI/EcoRI linearized pSIN-EF2-PuroR vector using In-Fusion^®^ HD Cloning kit (#639642, Clontech), respectively, to form pSIN-EpCAM and pSIN-EpICD vectors. To construct expression vector of pSIN-fEpCAM that contains ECFP-EpCAM fusion protein, ECFP gene was inserted into the downstream of EpCAM signal peptide and fused with EpCAM extracellular domain (EpEX) in vector pSIN-EpCAM. To construct expression vector pSIN-fEpCAM-mCherry that contains a mCherry protein in C-terminal downstream of EpICD, the fEpCAM was subcloned into pmCherry-N1, and the fragment of fEpCAM-mCherry was then subcloned into pSIN-EF2- PuroR vector. Expression vectors of *OCT4, SOX2, KLF4, c-MYC, NANOG, LIN28, SALL4*, and *ESRRB* from porcine iPSCs were constructed by pMXs vector, which were reported previously^9^. All constructs used in this study were listed in Table S2.

### Cell Reprogramming

For preparing retroviral particles, 2 × 10^6^ 293 T cells were planted on a 60 mm cultural dish. When cells reach to 80% confluence, pMXs vectors (4 μg/plasmid) carrying human *OCT4, SOX2, KLF4*, and *c-MYC* were transfected into 293 T cells together with 4 μg pCL-Eco and 2 μg pCMV-VSV-G at a ratio of 5:3:2 using Lipofectamine 2000 (Invitrogen). For preparing lentiviral particles, 4 μg shRNA constructs of pSIH (−), pSIH259i, pSIH409i, and pSIH727i and 4 μg EpCAM constructs of pSIN-EpCAM and pSIN-EpICD were transfected into 293 T cells with 4 μg pSPAX2 and 2 μg pMD 2.G, respectively. After 48 hours post-transduction, media with viral particles were collected and filtered through 0.45 μm filter (Millipore). To induce cell reprogramming, the equal amount of hOSKM viral supernatants mixed with 8 mg/mL Polybrene were infected into PEF cells for 12 h. To increase infection efficiency, a second infection was applied depending on the experimental needs. The infected PEF cells were then reseed at 2 × 10^4^ cell/cm^2^ and cultured in the iPS medium for 13 days.

### piPS Cell Differentiation

To detect the differentiation potential when knockdown or overexpression of EpCAM, DOX-iPSCs were transfected with viral particles that carrying either pSIN-EpCAM or pSIH-409i. After one passage, cells were detached from feeders by 1 mg/mL collagenase type IV and transferred to an ultra-low-attachment culture dish with the medium without growth factors and small molecules. For 3 days of suspension culturing, the embryoid bodies were formed, which were then transferred to a gelatine-coated culture dish and continually cultured in the regular medium (DMEM + 15% FBS) for 5 days. The spontaneously differentiated cells were harvested and used for RT-PCR analysis.

### Luciferase Assay

For the *EpCAM* promoter activity assay, PK-15 and PEF cells were planted on a 96-well plate. When cells reach to 80% confluence, 1 μg pGL3-EpCAM-pro and pGL3-Basic with 0.01 μg pRL-TK vector that is an internal control were co-transfected into cells, respectively. To determine *EpCAM* promoter activation, which was activated by human OSKM and porcine OCT4, SOX2, KLF4, c-MYC, NANOG, LIN28, SALL4, and ESRRB, 293 T cells were planted on a 96-well plate. When cells reach to 80% confluence, the mixed pMXs vectors (0.5 μg) with 0.5 μg pGL3-EpCAM-pro and 0.01 μg pRL-TK were co-transfected into cells, respectively.

To determine EpICD regulation function, PEF cells were planted on a 96-well plate. When cells reach to 80% confluence, 0.5 μg pGL3-OCT4-pro, pGL3-SOX2-pro, pGL3-LIN28-pro, and pGL3-ESRRB-pro with pSIN-EpICD, pSIN-EGFP, and 0.01 μg pRL-TK were co-transfected into PEFs, respectively. Transfection was performed using Lipofectamine 2000 (Invitrogen). To confirm EpICD signaling pathway, 3 μM CHIR99021 (a GSK3 inhibitor), 20 μM PS-341 (a proteasome inhibitor), and control DMSO were added to media at 12 hours post transfection, respectively. Cells were harvested at 36 h post transfection and lysed at room temperature for 10 min using passive lysis buffer (E1941, Promega). Luciferase activity was detected by luciferase assay reagents (E1960, Promega) in a 96-well solid white flat bottom microplate (#3917, Corning) using BHP9504 microplate luminometer (D04407H, Hamamatsu, China). Triplicates were measured for each treatment, and the average values of the ratio of firefly luciferase units to Renilla luciferase units were used for data analysis.

### Immunofluorescence Assay and Alkaline Phosphatase Staining

For immunofluorescence staining, cells were fixed with 4% paraformaldehyde in PBS for 15 min and blocked by 1% BSA at room temperature for 1 h. Cells were then incubated with primary anti-EpCAM antibody (1:200, ab71916, Abcam) at 4 °C overnight. After washed three times with PBS, cells were incubated with FITC conjugated secondary anti-rabbit antibody for 1 h. Nuclei were stained with 10 ug/mL Hoechst 33342 for 2 min. Images were captured under a Nikon fluorescence microscope. The alkaline phosphatase (AP) staining was determined by AST Fast Red TR and α-Naphthol AS-MX Phosphate (Sigma Aldrich) as our previously described^4^. Images were captured by a Pentax K-50 digital camera.

### Western Blotting

To determine endogenous EpCAM expression, PK-15 cells treated by shRNA for 60 h and PEF cells transfected with pSIN-EpCAM were lysed by RIPA buffer (Thermo Scientific) for 10 min on ice, resuspended in 5 × SDS-PAGE loading buffer (50 mM Tris-HCl pH 6.8, 2% SDS, 10% glycerol, 2% β-mercaptoethanol and 0.05% bromophenol blue), and heated at 100 °C for 5 min. A 15 μL cell lysate was loaded onto 10% SDS-PAGE gel. After electrophoresis, proteins were transferred to a PVDF membrane (LC2002, Invitrogen) by semidry electrophoretic transfer (Bio-Rad) for 45 min at 15 V. The membrane was blocked with blocking buffer (20 mM Tris/HCl pH7.6, 137 mM NaCl, 0.1% Tween 20, and 8% skim milk) at 25 °C for 2 h, and then incubated with the primary anti-EpCAM antibody (1: 1000, ab71916, Abcam) in the blocking buffer at 4 °C overnight. After washing three times with TBS-T buffer (20 mM Tris/HCl pH 7.6, 137 mM NaCl, 0.1% Tween 20), the membrane was incubated with HRP-conjugated secondary anti-rabbit antibody (1:2000) at room temperature for 1 h. After washing three times in TBS-T at room temperature, the membrane was incubated in the enhanced chemiluminescent substrate (#32106, Pierce) and detected with a Chemiluminescent Imaging System (ZY058176, Tanon-4200, China). To monitor the fEpCAM-mCherry fusion protein cleavage, PK-15 cells were transduced with pSIN-fEpCAM-mCherry for 12 h, and then TAPI-1 and DAPT was added to media. At 48 h post transduction, cells were directly lysed by RIPA buffer for 10 min on ice, and protein blotting were performed as mentioned above with anti-mCherry antibody (1:1000, KM8017, Sungene Biotech, China) and anti-EpCAM antibody (1:1000), respectively. The HRP-conjugated secondary anti-mouse antibody (1:2000) was applied following with the incubation with the enhanced chemiluminescent substrate. To verify the EpICD expression, PEF cells were transduced with pSIN-EpICD for 12 h, and then PS-341 was added to medium. Cells were harvested at 60 h post transduction. The anti-His antibody (1:1000, #12698, Cell Signaling) were used to detect EpCAM-His fusion protein. To dissect the beta-catenin and EpICD signaling, PEF cells treated with 3 μM CHIR99021 or 20 μM PS-341 for 24 h were harvested, and nuclear proteins were extracted by the Nuclear Protein Extraction kit (R0050, Solarbio, China). The anti-beta-catenin antibody (1:1000, ab23671, Abcam) was applied following above procedure. The anti-beta-actin antibody (1:2000, KM9001, Sungene Biotech, China) and anti-histone H3 antibody (1:1000, #5192 Cell Signaling) were used as internal controls for the total proteins and nuclear proteins, respectively.

### Bioinformatic Analysis

The microarray data were acquired from GEO database as previously described^13^, and GEO numbers are: GSE48434 for PEF^4^,^42^, PS24^4^, 30 AC5^4^, iPF4-2^42^, and piPSCs-w^2^, GSE26369 for pESK^7^, and GSE15472 for IC1, ID4, and ID6^43^. Pluripotent genes, lineage specific genes, and MET (mesenchymal-epithelial transition)/EMT (epithelial–mesenchymal transition) markers were chosen for this study. R software for windows was used to draw the heatmap. Sequencing data of mRNA from porcine early stage embryo were acquired from the Lab Archive (www.ncbi.nlm.nih.gov/sra) under accession number SRA076823 as described previously^44^, and the pluripotency related genes were chosen and charted using GraphPad Prism™ 5.0 for windows.

### Statistical analysis

Statistical analyses were performed with the SPSS 16.0 for windows. Two-way ANOVA were used to study differences between grouped data, Student’s T tests were performed with one way analysis. Statistical significance was accepted at *P* < 0.05.

## Additional Information

**How to cite this article**: Yu, T. *et al*. EpCAM Intracellular Domain Promotes Porcine Cell Reprogramming by Upregulation of Pluripotent Gene Expression via Beta-catenin Signaling. *Sci. Rep.* **7**, 46315; doi: 10.1038/srep46315 (2017).

**Publisher's note:** Springer Nature remains neutral with regard to jurisdictional claims in published maps and institutional affiliations.

## Supplementary Material

Supplementary Materials
