# Supplementary material for: EpCAM Intracellular Domain Promotes Porcine Cell Reprogramming by Upregulation of Pluripotent Gene Expression via Beta-catenin Signaling
Source: Sci Rep. 2017 Apr 10;7:46315. doi: 10.1038/srep46315 (PMC5385527; doi:10.1038/srep46315)
Supplement: Supplementary Materials [file srep46315-s1.pdf]

SUPPORTING INFORMATION

EpCAM Intracellular Domain Promotes Porcine Cell Reprogramming by Upregulation of Pluripotent Gene Expression via Beta-catenin Signaling

Running title: EpCAM promotes porcine cell reprogramming

Tong Yu[§],, Yangyang Ma[§],, Huayan Wang*

Department of Animal Biotechnology, College of Veterinary Medicine, Northwest A&F University, Yangling, Shaanxi 712100, China

§ These authors contributed equally to this work.

* Correspondence and requests for materials should be addressed: Huayan Wang, Department of Animal Biotechnology, College of Veterinary Medicine, Northwest A&F University, Yangling, Shaanxi 712100, China. Telephone: 86-029-87080069; fax: 86-029-87080068; E-mail: hhwang101@163.com or hhwang101@nwsuaf@edu.cn.

Supplementary Tables

Table S1 List of primers used in this study

Primer Names	Primer Sequences	Genes Name
**pc-EpCAM-Clong-977-F**	CCCaagcttGGGATGGCGCCCCCCCAGGT	EPCAM
**pc-EpCAM-Clong-977-R**	CCGctcgagCGGTTACGGTGTTATGCATTGAGTTCCCTAT	
**EPCAM-HIS-in-fusion-1014-F**	gtgtcgtgaggaattcATGGCGCCCCCCCAGGTCCT	EPCAM-HIS-tag
**EPCAM-HIS-in-fusion-1014-R**	agatgcatgcggatctcAGTGGTGATGGTGATGATGCGCATTGAGTTCCCTATGCATCT	
**EPICD-HIS-in-fusion-151-F**	gtgtcgtgaggaattcATGTCCACGAAGAAAAGAAGGGCA	EPICD-HIS-tag
**EPICD-HIS-in-fusion-151-R**	agatgcatgcggatctcAGTGGTGATGGTGATGATGCGCATTGAGTTCCCTATGCATCT	
**T-EPCAM-linear-3991-57-F**	GGCCGCGGCCACCGC	EPCAM split up
**T-EPCAM-linear-3991-57-R**	CAACAAGGATGTGTGTGTGAAAACTA	
**ECFP-in-fusion-746-58-F**	gcggtggcgcggccATGGTGAGCAAGGGCGAG	ECFP insertion
**ECFP-in-fusion-746-58-R**	cacacatccttgttgCTTGTACAGCTCGTCCATGCC	
**fEPCAM-HIS-in-fusion-1748-F**	gtgtcgtgaggaattcATGGCGCCCCCCCAGGTCCT	f-EPCAM-HIS-tag
**fEPCAM-HIS-in-fusion-1748-R**	agatgcatgcggatctcAGTGGTGATGGTGATGATGCGCATTGAGTTCCCTATGCATCT	
**EPCAM-mCherry-in-fusion-2441-F**	gtgtcgtgaggaattcATGGCGCCCCCCCAGGTCCT	f-EPCAM-mCherry
**EPCAM-mCherry-in-fusion-2441-R**	agatgcatgcggatccCTACTTGTACAGCTCGTCCATGCCGCCGGT	
**pc-POU5F1-HindIII-1083-F**	CaagcttATGGCGGGACACCTGGCT	pMXs-pOCT4
**pc-POU5F1-Xoh1-1083-R**	GctcgagTCAGTTTGAATGCATGGGGGA	
**pc-SOX2-HindIII-960-F**	CaagcttATGTACAACATGATGGAGACGGA	pMXs-pSOX2
**pc-SOX2-XhoI-960-R**	GctcgagTCACATGTGAGAGAGAGGCAGTG	
**pc-KLF4-HindIII-1461+F**	CCCaagcttATGAGGCAGCCACCTGGCGA	pMXs-pKLF4
**pc-KLF4-XhoI-1461+R**	CCGctcgagTTAAAAATGCCTCTTCATGTGTAAGGCAA	
**pc-cMyc-HindIII-1320+F**	CCCaagcttATGCCCCTCAACGTCAGCTTCA	pMXs-pC-MYC
**pc-cMyc-XhoI-1320+R**	CCGctcgagTTATGGGCAAGAGTTCCGTAGCTGT	
**pc-NANOG-HindIII-929-F**	CaagcttATGAGTGTGGATCCAGCTT	pMXs-pNANOG
**pc-NANOG-Xho1-929-R**	GctcgagTCACATATCTTCAGGCTGTA	
**pc-LIN28a-HindIII-618-F**	CaagcttATGGGCTCTGTGTCAAACCAG	pMXs-pLIN28
**pc-LIN28a-Xho1-618-F**	GctcgagTCAATTCTGAGCCTCTGGGAG	
**pc-SALL4a,b-HindIII-3153,1851-F**	CaagcttATGTCGAGGCGCAAGCAG	pMXs-pSALL4
**pc-SALL4a,b-Xho1-3153,1851-R**	GctcgagTCAGCTGACCGCGATCTTG	
**pc-ESRRB-BamHI-1358-F**	TAAggatccATGTCCGCAGACGACCGGCA	pMXs-pESRRB
**pc-ESRRB-XhoI-1358-R**	GctcgagTCAGACCTTGGCCTCCAGCAT	
**mCherry-HindIII-723-F**	CaagcttATGGTGAGCAAGGGCGA	pMXs-mCherry
**mCherry-XhoI-723-F**	GctcgagCTACTTGTACAGCTCGTCCA	
**EPCAM-pro-3819-F**	aggtaccgagctcttaCGCGTGGGTTTGCTGAGT	EPCAM promoter
**EPCAM-pro-3819-R**	gagatatctcgagcgtcgacGGTGCGCGGGCGCCGA	
**OCT4-pro-4191-F**	aggtaccgagctcttaTGCCAAGGAGAGGGAGCT	OCT4 promoter
**OCT4-pro-4191-R**	gagatatctcgagcgtcgacGGGGAAGGAAGGCGCC	
**SOX2-pro-2991-F**	aggtaccgagctcttaTGCTATTAAGCGCGGACCAA	SOX2 promoter
**SOX2-pro-2991-R**	gagatatctcgagcgtcgacGCGGGCGCTGTGCG	
**LIN28-pro-3251-F**	aggtaccgagctcttaTCCAGGTCCTTAGGGAGCAA	LIN28 promoter
**LIN28-pro-3251-R**	gagatatctcgagcgtcgacGGTAGTCGGCTGAGCC	

**ESRRB-pro-3337-F**	aggtaccgagctcttaAAGCCTGCACCTGCCAG	ESRRB promoter
**ESRRB-pro-3337-R**	gagatatctcgagcgtcgacTCTGTTCAGCAGCTGGTTGTGGTAGCCGAGAGGGTC	
**pc-GAPDH-P-422-F**	TTCACGACCATGGAGAAGGC	GAPDH
**pc-GAPDH-P-422-R**	ACCGACACGTTGGCAGTAG	
**pc-ACTB-Q-161-F**	GTGACAGCAGTCGGTTGGAT	beta-ACTIN
**pc-ACTB-Q-161-R**	TTTTGGGAAGGCAGGGACTT	
**pc-EpCAM-Q-178-F**	CTTGGTCAATGCCAGTGTACTT	EPCAM
**pc-EpCAM-Q-178-R**	TCTCGTCACAGTCAGGATCATAG	
**pc-EpCAM-P-483-F**	TGAACACTGCTGGGGTCAGAA	EPCAM
**pc-EpCAM-P-483-R**	CAATCGCTATCGCCACAACTG	
**pc-POU5F1-Q-179-F**	TGAGGCTTTGCAGCTCAGTT	OCT4
**pc-POU5F1-Q-179-R**	TCTCCAGGTTGCCTCTCACT	
**pc-SOX2-Q-219-F**	CATGAAGGAGCACCCGGATT	SOX2
**pc-SOX2-Q-219-R**	ATCATGCTGTAGCTGCCGTT	
**pc-LIN28A-Q-246-F**	TGCCGGCATCTGTAAATGGT	LIN28
**pc-LIN28A-Q-246-R**	CTCTCGCTCCCAATGCAGAA	
**pc-SALL4A-Q-217-F**	CCCCAACACATCAACTCGGA	SALL4
**pc-SALL4A-Q-217-R**	ACTCGGCACAGCATTTCTCA	
**pc-ESRRB-Q-149-F**	ATGCCTCAAAGTGGGGATGC	ESRRB
**pc-ESRRB-Q-149-R**	TTTTAGTCAATGGCTTCTTCGCA	
**pc-ADAM17-P-529-F**	GTGTTTTCAAGAGCGCAGCA	ADAM17
**pc-ADAM17-P-529-R**	TCGCTTCTCACACTTGCCAT	
**pc-PSEN2-P-432-F**	TGTACGATCTCGTGGCTGTG	PSEN2
**pc-PSEN2-P-432-R**	GCAGGAGGGTCAGACACAAA	
**pc-CTNNB1-P-464-F**	TGGACAATGGCTACCCAAGC	CTNNB1
**pc-CTNNB1-P-464-R**	ATTGCACGTGTGGCAAGTTC	
**pc-GSK3B-P-186-F**	CGAGACACACCTGCACTCTT	GSK3B
**pc-GSK3B-P-186-R**	TGACGCAGAAGCGGTGTTAT	
**pc-FHL2-P-P-767-F**	GGATGCGACTGCAAGGACTTA	FHL2
**pc-FHL2-P-P-767-R**	GCGGTTTGGAGGAGAATCCA	
**pc-LEF1-P-535-F**	ATCCCATCACGGGAGGATT	LEF1
**pc-LEF1-P-535-R**	GCAGCTGTCATTCTTGGACCT	
**pc-NESTIN-Q-216-F**	**CGCCATTGTCCCCATCCGT**	**NESTIN**
**pc-NESTIN-Q-216-R**	CAGCTCATTCTGCTCCTCTAGC	
**pc-CD34-Q-71-F**	**TGGGCATCACTGGCTACTTG**	**CD34**
**pc-CD34-Q-71-R**	GTCTTCGCCCAGCCTTTCT	
**pc-FOXA1-Q-196-F**	**CCTCAGAGTTGAAGACTCCAG**	**FOXA1**
**pc-FOXA1-Q-196-R**	TGCTCAGAGGAGGACATGAG	

Table S2 List of vectors used in this study

Name	Function	Genes	Promoter
**pMXs-EGFP**	Retro-virus / Expression	EGFP	Virus
**pMXs-hOCT4**	Retro-virus / Expression	Human OCT4	Virus
**pMXs-hSOX2**	Retro-virus / Expression	Human SOX2	Virus
**pMXs-hKLF4**	Retro-virus / Expression	Human KLF4	Virus
**pMXs-hC-MYC**	Retro-virus / Expression	Human C-MYC	Virus
**pMXs-pOCT4**	Retro-virus / Expression	Porcine OCT4	Virus
**pMXs-pSOX2**	Retro-virus / Expression	Porcine SOX2	Virus
**pMXs-pKLF4**	Retro-virus / Expression	Porcine KLF4	Virus
**pMXs-pC-MYC**	Retro-virus / Expression	Porcine C-MYC	Virus
**pMXs-pNANOG**	Retro-virus / Expression	Porcine NANOG	Virus
**pMXs-pLIN28**	Retro-virus / Expression	Porcine LIN28	Virus
**pMXs-pSALL4**	Retro-virus / Expression	Porcine SALL4	Virus
**pMXs-pESRRB**	Retro-virus / Expression	Porcine ESRRB	Virus
**pMXs-pNANOG**	Retro-virus / Expression	Porcine NANOG	Virus
**pRL-TK**	Reporter / Dual luc-assay	*Renilla reniformis*	HSV TK
**pGL3-Basic**	Reporter / Dual luc-assay	Firefly luciferase	None
**pGL3-EPCAM-Pro**	Reporter / Dual luc-assay	Firefly luciferase	EPCAM
**pGL3-OCT4-Pro**	Reporter / Dual luc-assay	Firefly luciferase	OCT4
**pGL3-SOX2-Pro**	Reporter / Dual luc-assay	Firefly luciferase	SOX2
**pGL3-LIN28-Pro**	Reporter / Dual luc-assay	Firefly luciferase	LIN28
**pGL3-ESRRB-Pro**	Reporter / Dual luc-assay	Firefly luciferase	ESRRB
**pTRIP-CAGG-EGFP**	Lenti-virus / Reporter	EGFP / PuroR	CAGG
**pTRIP-EPCAM-EGFP**	Lenti-virus / Reporter	EGFP / PuroR	EPCAM
**pSIH-(-)**	Lenti-virus / shRNA	None / copGFP	H1 / CMV
**pSIH-EPCAM-259i**	Lenti-virus / shRNA	EPCAM shRNA-259 / copGFP	H1 / CMV
**pSIH-EPCAM-409i**	Lenti-virus / shRNA	EPCAM shRNA-409 / copGFP	H1 / CMV
**pSIH-EPCAM-727i**	Lenti-virus / shRNA	EPCAM shRNA-727 / copGFP	H1 / CMV
**pSIN-EF2-EGFP**	Lenti-virus / Expression	EGFP / PuroR	EF-1a
**pSIN-EF2-EPCAM**	Lenti-virus / Expression	EPCAM-HIS-tag / PuroR	EF-1a
**pSIN-EF2-EPICD**	Lenti-virus / Expression	EPICD-HIS-tag / PuroR	EF-2a
**pSIN-EF2-fEPCAM**	Lenti-virus / Expression	fEPCAM-HIS-tag / PuroR	EF-1a
**pSIN-EF2-fEPCAM-mCh**	Lenti-virus / Expression	fEPCAM-mCherry / PuroR	EF-1a

Supplementary Figures

A

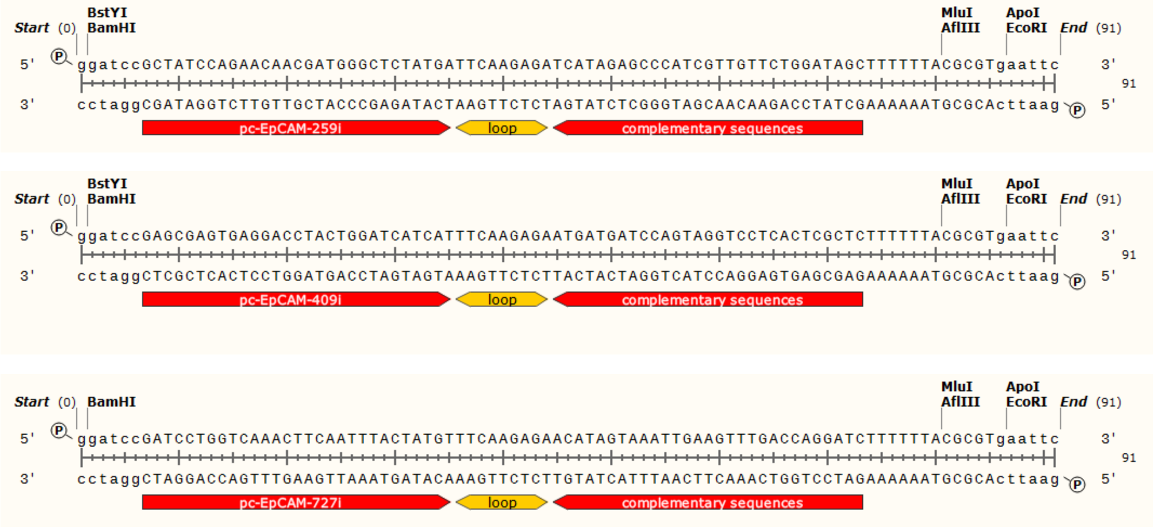

B

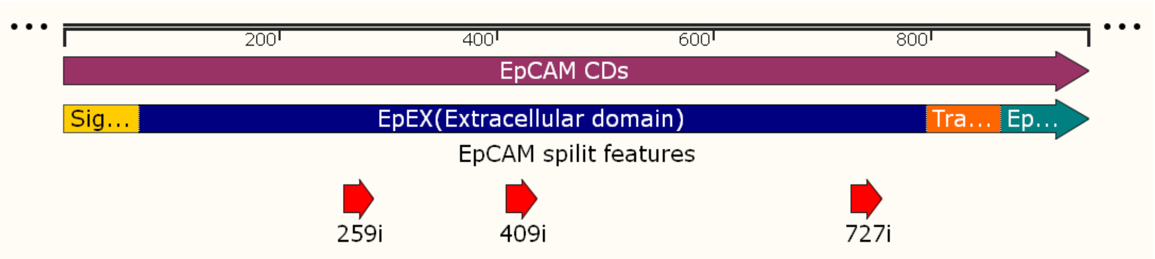

Figure S1. EpCAM shRNA Sequences. A, DNA sequences of shRNA259, shRNA409, and shRNA727. **B,** Schematic diagram of shRNA locations (red arrows) in *EpCAM* CDS.

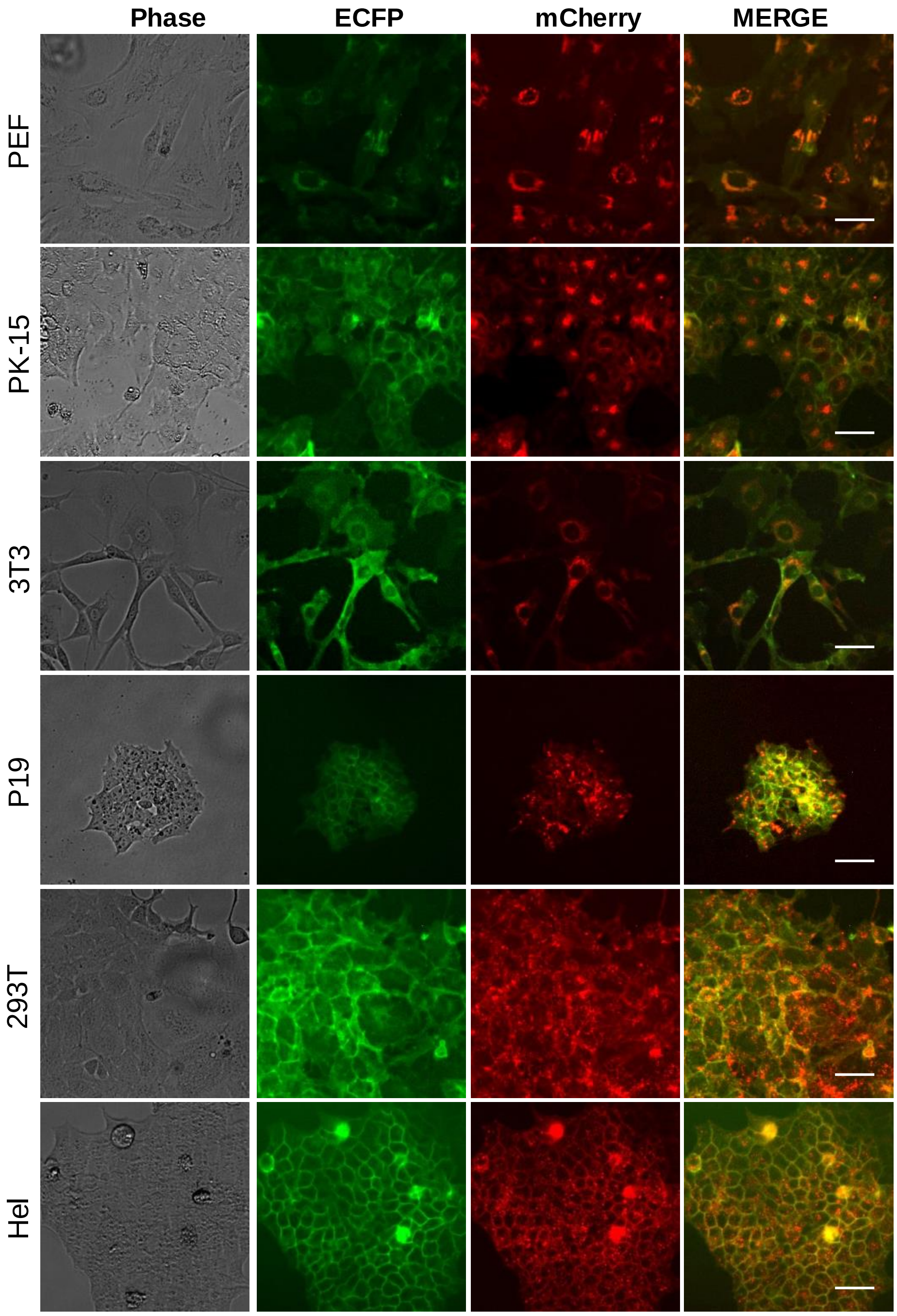

Figure S2. EpCAM expression in different cell lines. Cell lines from pig (PEF and PK-15), mouse (NIH3T3 and P19), and human (293T and Hela) were transfected by lentiviral construct pSIN-fEpCAM-mCherry for 48 h. Translocation of fusion proteins fEpCAM-mCherry (yellow) and EpICD-mCherry (red) were visualized under the fluorescence microscope. Scale bar, 50 μm.
